# Successor to the Late Dr. Campbell

**Published:** 1888-10

**Authors:** 


### *SUCCESSOR TO THE LATE DR. CAMPBELL.*

The Trustees of Niagara University, on the recommendation of the Medical Faculty, have elected Dr. Albert E. Persons Professor of Materia Medica and Therapeutics, in place of Dr. F. R. Campbell, deceased. One year ago, Dr. Persons was appointed Lecturer on Hygiene, and the position has been filled with so much ability that we regard the promotion as a merited recognition of his fitness for the broader field of labor to which he is called.

This chair has been previously occupied by able men—Drs. Stockton and Campbell. The compliment tendered to the new incumbent is a flattering tribute to his professional attainments, and to his fitness for this most important department of medicine. Untiring zeal and patient industry have brought success to Dr. Persons as a teacher of Preventive Medicine. This preparation for the position of Professor of Materia Medica and Therapeutics, in which the curative properties of drugs are taught, will bring success, and make him a strong and valuable addition to the Faculty.

We congratulate the new Professor on the honor bestowed upon him, and the Trustees and Faculty on the wisdom of their choice.

We understand that the Physicians' Club has decided to reorganize under the name of the Buffalo Pathological and Clinical Society, the object being to limit the work in accordance with the new title. We think this is a good move, and that it will improve the usefulness of the club. We feel sure that the society will take an advanced place among the various medical associations of the city.

